# Oxidative Stress, Inflammation, and Altered Lymphocyte E-NTPDase Are Implicated in Acute Dyslipidemia in Rats: Protective Role of Arbutin

**DOI:** 10.3390/ph17101343

**Published:** 2024-10-08

**Authors:** Reem S. Alruhaimi, Omnia E. Hussein, Sulaiman M. Alnasser, Mousa O. Germoush, Meshal Alotaibi, Emad H. M. Hassanein, Mohamed El Mohtadi, Ayman M. Mahmoud

**Affiliations:** 1 Department of Biology, College of Science, Princess Nourah bint Abdulrahman University, Riyadh 11671, Saudi Arabia; 2 Higher Technological Institute for Applied Health Sciences, Beni-Suef 62764, Egypt; 3 Department of Pharmacology and Toxicology, College of Pharmacy, Qassim University, Buraydah 51452, Saudi Arabia; 4 Biology Department, College of Science, Jouf University, Sakakah 72388, Saudi Arabia; 5 Department of Pharmacy Practice, College of Pharmacy, University of Hafr Albatin, Hafar Al Batin 39524, Saudi Arabia; 6 Department of Pharmacology & Toxicology, Faculty of Pharmacy, Al-Azhar University-Assiut Branch, Assiut 71524, Egypt; 7 Department of Biology, Edge Hill University, Ormskirk L39 4QP, UK; 8 Department of Life Sciences, Faculty of Science and Engineering, Manchester Metropolitan University, Manchester M1 5GD, UK; 9 Molecular Physiology Division, Zoology Department, Faculty of Science, Beni-Suef University, Beni-Suef 62514, Egypt

**Keywords:** dyslipidemia, arbutin, inflammation, oxidative stress

## Abstract

Background/Objectives: Dyslipidemia is frequently linked to various disorders, and its clinical relevance is now recognized. The role of inflammation and oxidative stress (OS) in dyslipidemia has been acknowledged. This study assessed the potential of arbutin (ARB) to prevent dyslipidemia and its associated OS and inflammation in rats with acute hyperlipidemia. Methods: Rats received ARB orally for 14 days and a single intraperitoneal injection of poloxamer-407 on day 15. Results: Poloxamer-407 elevated circulating cholesterol (CHOL), triglycerides (TG), very low-density lipoprotein (vLDL), and LDL, and reduced high-density lipoprotein (HDL)-C and lipoprotein lipase (LPL). ARB ameliorated the circulating lipids and LPL, and suppressed 3-hydroxy-3-methylglutaryl CoA reductase (HMGCR) in rat liver and in vitro. Fatty acid synthase (FAS) in rat liver and its in vitro activity were suppressed by ARB, which also upregulated the LDL receptor (LDL-R) and ABCA1, and had no effect on ABCG5 and ABCG8 mRNA. ARB ameliorated liver malondialdehyde and nitric oxide and enhanced antioxidants in rats with dyslipidemia. Liver NF-κB p65 and blood inflammatory cytokines were increased in dyslipidemic rats, effects that were reversed by ARB. Moreover, ARB effectively suppressed lymphocyte E-NTPDase and E-ADA activities in dyslipidemic rats. The biochemical findings were supported by in silico data showing the affinity of ARB to bind LDL-R PCSK9 binding domain, HMGCR, FAS, and E-NTPDase. Conclusions: ARB possessed anti-dyslipidemia, anti-inflammatory, and antioxidant effects mediated via the modulation of CHOL and TG synthesis, LPL, lymphocyte E-NTPDase and E-ADA, and cytokine release in rats. Thus, ARB could be an effective agent to attenuate dyslipidemia and its associated OS and inflammation, pending further studies as well as clinical trials.

## 1. Introduction

Dyslipidemia refers to changes in circulating lipid profile and are frequently linked to various clinical disorders [1]. The adoption of sedentary lifestyle, fast food, and unhealthy dietary habits along with rapid economic growth are implicated in the development of dyslipidemia in different countries [1]. Dyslipidemia involves the presence of abnormal levels of plasma cholesterol (CHOL), triglycerides (TG) and their associated lipoproteins [2]. Elevated blood low-density lipoprotein (LDL)-cholesterol (LDL-C) is among the risk factors of cardiovascular disease (CVD), whereas hypercholesterolemia represents the most prevalent type of dyslipidemia [1]. Given the increased prevalence of dyslipidemia and other factors implicated in the development of CVD, in 2019, it was reported that the number of patients had reached 523 million and that the number of deaths had risen to 18.6 million [3]. Elevated LDL-C is a significant cause of ischemic stroke and ischemic heart disease (IHD) in both developing and developed countries [1,2]. High CHOL is associated with atherosclerosis pathogenesis and its subsequent IHD, myocardial infarction (MI), and other CVDs [2]. Statins are the first-line therapeutic approach to treat dyslipidemia and significantly reduce CVD mortality. Statins are highly effective and safe for use in the vast majority of patients [1]. However, the long-term use of statins might increase the risk of adverse effects in patients with multiple medical co-morbidities [4]. Therefore, the development of new lipid-lowering agents from natural sources could be of great value from the safety and accessibility points of view.

TG and CHOL are derived from hepatic synthesis and dietary sources. Fatty acids (FAs) found in the diet are taken by transport proteins on the enterocytes for the synthesis of TG [5], whereas hepatic synthesis is the primary source of CHOL [6]. The dietary sources represent 15–20% of CHOL, which is absorbed in the upper small intestine cells [7]. Within the liver, de novo biosynthesis of CHOL is mediated via the activity of 3-hydroxy-3-methylglutaryl CoA reductase (HMGCR) [6]. CHOL could also be taken from lipoprotein uptake by hepatocytes and free CHOL converted into esters (CE) for transit in lipoproteins along with TG [8]. Both hepatic and intestinal packaging of CE and TG into lipoproteins are facilitated by microsomal triglyceride transfer protein [9]. Both the synthesis and degradation of lipids are controlled by several proteins, including HMGCR and LDL receptor (LDL-R) [10] and pathways of CHOL efflux [11,12]. The reverse CHOL transport (RCT) and direct biliary excretion mediated via ABC subfamily A member 1 (ABCA1) and ABCG5/8, respectively, maintain CHOL homeostasis [11,12].

Elevated blood lipids are linked to some pathologic processes, including oxidative stress (OS) and systemic inflammation [13,14,15]. Redox imbalance induced by high levels of reactive oxygen species (ROS) and diminished antioxidants is central in the pathophysiology of many CVDs and dyslipidemia is associated with elevated ROS and OS [13,16]. In atherosclerosis, for instance, the excess generation of intracellular ROS mediated via lipid accumulation contributes to chronic inflammation and cell dysfunction [15,17]. Dyslipidemia-provoked excess ROS can oxidize LDL, DNA, and many proteins and activate nuclear factor (NF)-κB, resulting in inflammation, mitochondrial dysfunction, and cell injury [13,18]. The provoked inflammatory response can influence alterations in lipid metabolism as shown in diabetes, obesity, and atherosclerosis [19,20]. Hence, agents that exhibit antioxidant, anti-inflammatory and lipid-lowering efficacies can represent effective candidates for the management of hyperlipidemia and preventing its associated CVD.

**Figure 1:**
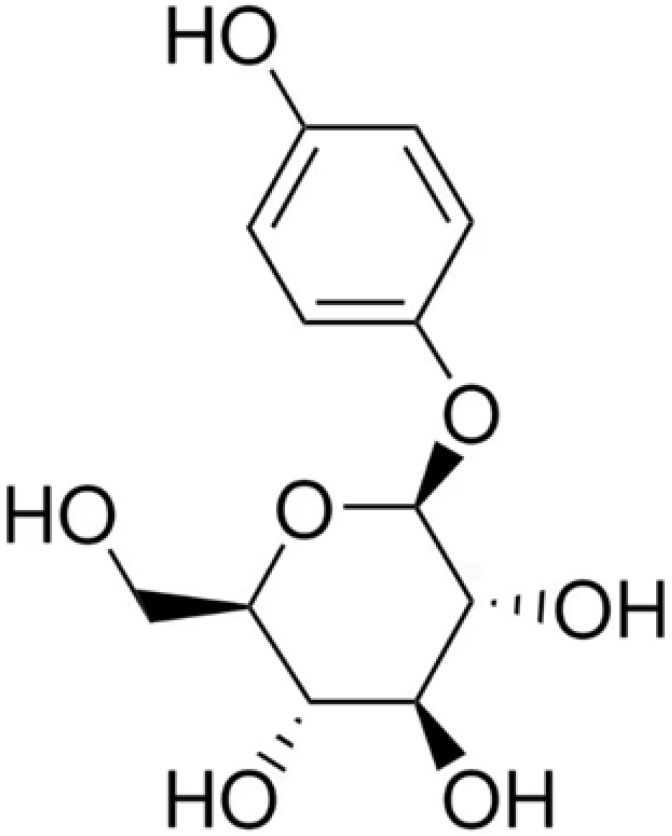
Chemical structure of arbutin (hydroquinone O-*β*-D-glucopyranoside).

Numerous plant-derived compounds have demonstrated beneficial effects against dyslipidemia, OS, and inflammation in preclinical models [21,22,23,24,25]. Among natural products, polyphenolics have multiple health-promoting and pharmacological effects due to possessing antioxidant, anti-inflammatory, and lipid-lowering properties [26,27,28]. The hydroquinone glucoside arbutin (ARB) (Figure 1), a bioactive component of bearberry and other plants, is widely employed as anti-pigmentation agent in skincare products [29]. It showed other beneficial effects, most importantly anti-inflammatory, antioxidant, hepatoprotective, and nephroprotective [30,31,32]. Research from our lab and others revealed the anti-diabetic potential of ARB in rodents with type 1 [33] and type 2 diabetes [22]. In diabetes, ARB ameliorated hyperglycemia, OS, and inflammation [22,33]. The reported beneficial effects of ARB pinpointed its potential as an effective candidate in preventing dyslipidemia and its associated OS and inflammatory response. Therefore, this study evaluated its effect on dyslipidemia, OS, and inflammation in a rodent model of acute dyslipidemia induced by poloxamer-407 (P-407).

## 2. Results

### 2.1. ARB Alleviates P-407-Induced Dyslipidemia

**Figure 2:**
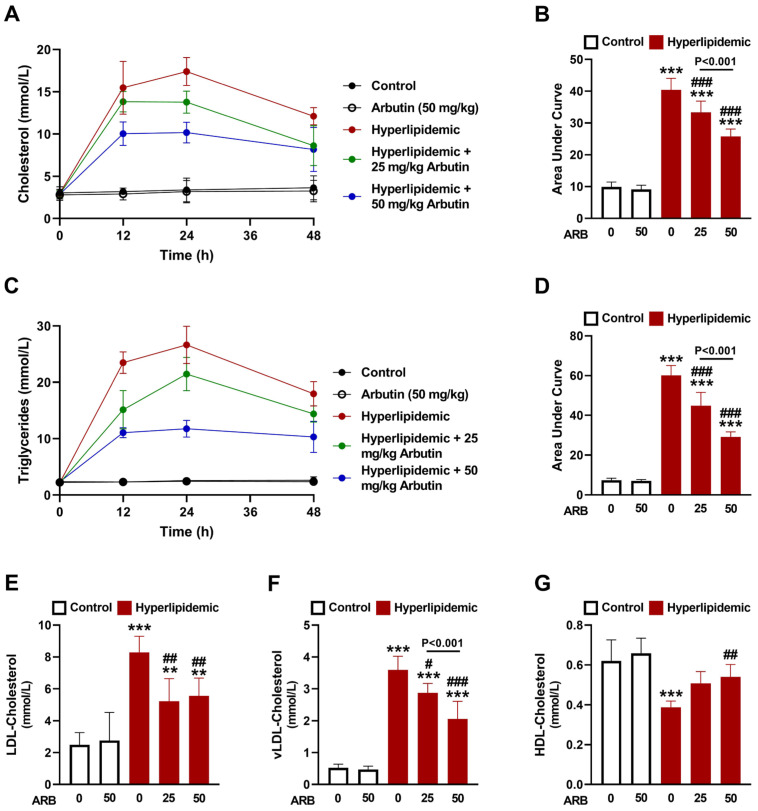
ARB decreased plasms CHOL (**A**,**B**) and TG (**C**,**D**) levels at 12, 24, and 48 h, and decreased LDL-C (**E**) and vLDL-C (**F**) and increased HDL-C (**G**) at 48 h in hyperlipidemic rats. Data are mean ± SEM (*n* = 6). ** *p* < 0.01 and *** *p* < 0.001 vs. Control. ^#^ *p* < 0.05, ^##^ *p* < 0.01, and ^###^ *p* < 0.001 vs. Hyperlipidemic.

P-407 caused hypercholesterolemia and hypertriglyceridemia marked by elevated plasma TC (Figure 2A,B) and TG (Figure 2C,D) after 12, 24, and 48 h (*p* < 0.001). ARB effectively and dose-dependently decreased TC and TG (*p* < 0.001). The determination of LDL-C (Figure 2E) and vLDL-C (Figure 2F) revealed remarkable elevation in P-407-administered animals, whereas HDL-C (Figure 2G) was significantly reduced after 48 h. ARB decreased LDL-C and vLDL-C, and enhanced HDL-C in P-407-administered rats, and its effect on vLDL-C was dose-dependent. However, ARB did not affect all assayed lipids when administered to normal rats.

### 2.2. Effect of ARB on LDL-R, ABCA1, and ABCG5/8 in Dyslipidemic Rats

**Figure 3:**
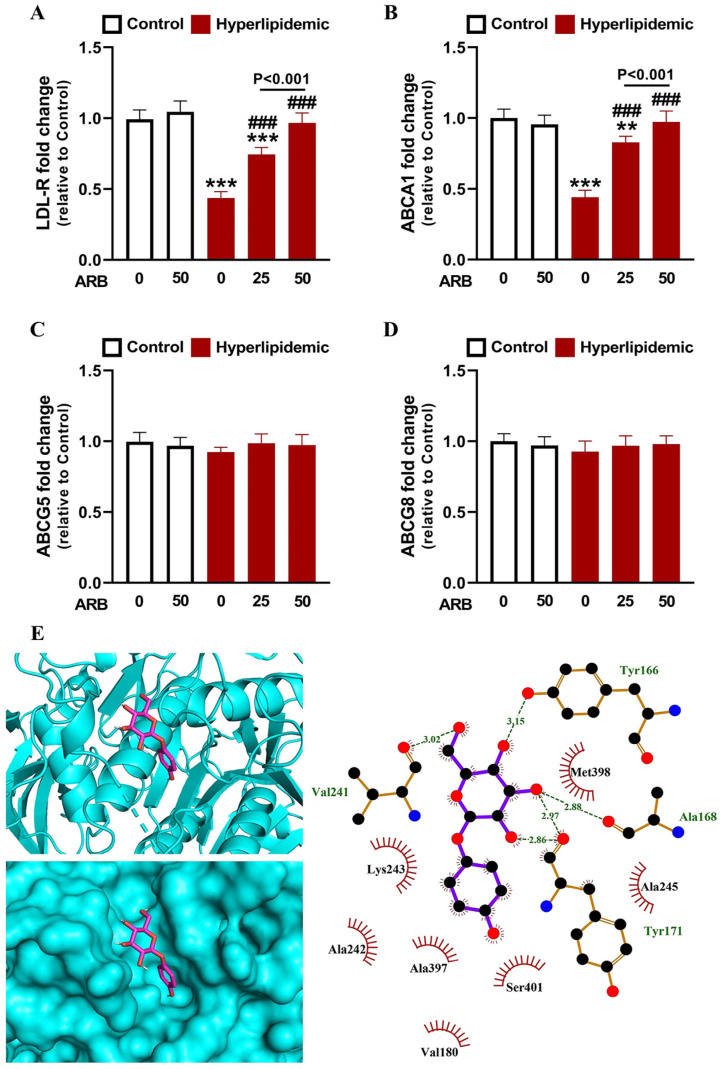
ARB upregulated LDL-R (**A**) and ABCA1 (**B**) mRNA and showed no effect on ABCG5 (**C**) and ABCG8 (**D**) mRNA in liver of hyperlipidemic rats. Data are mean ± SEM (*n* = 6). ** *p* < 0.01 and *** *p* < 0.001 vs. Control and ^###^ *p* < 0.001 vs. Hyperlipidemic. (**E**) Molecular docking of ARB with LDL-R PCSK9 binding domain showing the crystal structure and amino acid residues involved in polar bonding and hydrophobic interactions.

Changes in the mRNA levels of LDL-R (Figure 3A) and ABCA1 (Figure 3B) revealed a remarkable downregulation in the liver of dyslipidemic rats (*p* < 0.001). ARB promoted a dose-dependent increase in liver LDL-R and ABCA1 mRNA abundance in rats with dyslipidemia, whereas it had no effect in normal rats. ABCG5 (Figure 3C) and ABCG8 (Figure 3D) mRNA levels were not changed between groups (*p* > 0.05). The affinity of ARB to bind LDL-R proprotein convertase subtilisin/kexin type 9 (PCSK9) binding domain was explored in silico, and the data showed three polar bonding and seven hydrophobic interactions between ARB and LDL-R amino acid residues (Figure 3E, Table 1).

### 2.3. ARB Inhibits HMGCR Activity in Dyslipidemic Rats and In Vitro

**Figure 4:**
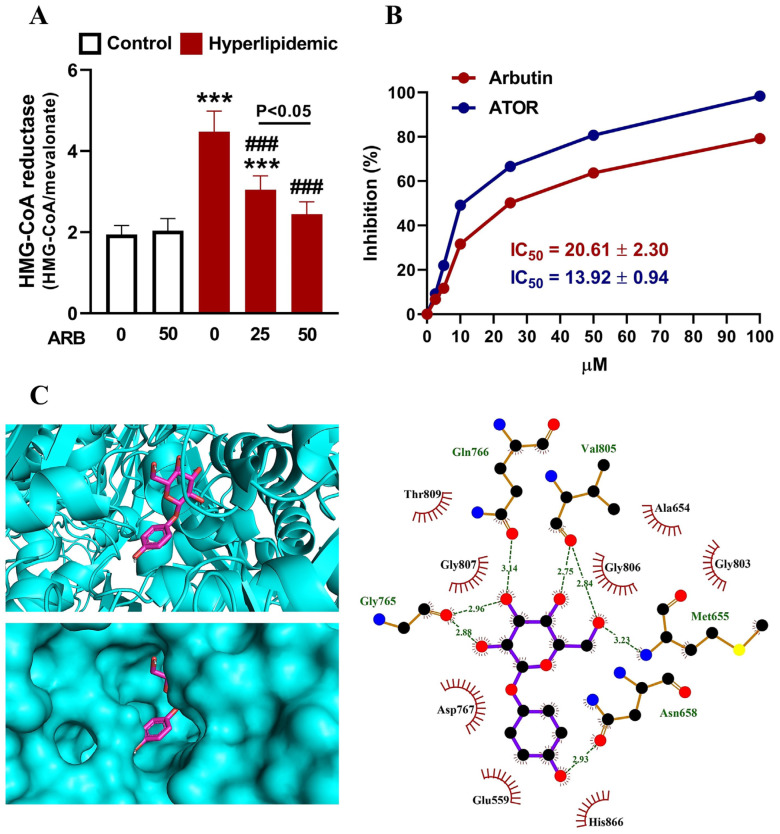
ARB suppressed HMGCR activity in the liver of hyperlipidemic rats (**A**). Data are mean ± SEM (*n* = 6). *** *p* < 0.001 vs. Control, and ^###^ *p* < 0.001 vs. Hyperlipidemic. (**B**) In vitro HMGCR inhibition activity of ARB and atorvastatin. Data are mean ± SEM (*N* = 3). (**C**) Molecular docking of ARB with HMGCR showing the crystal structure and amino acid residues involved in polar bonding and hydrophobic interactions.

Dyslipidemia induced by P-407 was associated with elevated HMGCR activity in the liver of rats significantly when compared with normal rats (*p* < 0.001; Figure 4A). Treatment with ARB did not affect HMGCR activity in normal rats but showed dose-dependent inhibition in the liver of rats with dyslipidemia. The HMGCR inhibitory activity of ARB was further investigated in vitro (Figure 4B) and in silico (Figure 4C). ARB showed the concentration-dependent inhibition of HMGCR with an IC_50_ value of 20.61 ± 2.30 µM, and atorvastatin (ATOR) exhibited an IC_50_ value of 13.92 ± 0.94 µM. In silico data showed the affinity of ARB to interact with five amino acid residues of HMGCR via polar bonding and eight residues via hydrophobic interactions (Table 1).

### 2.4. Effect of ARB on Lipoprotein Lipase (LPL) and Fatty Acid Synthase (FAS) in Rats with Dyslipidemia

**Figure 5:**
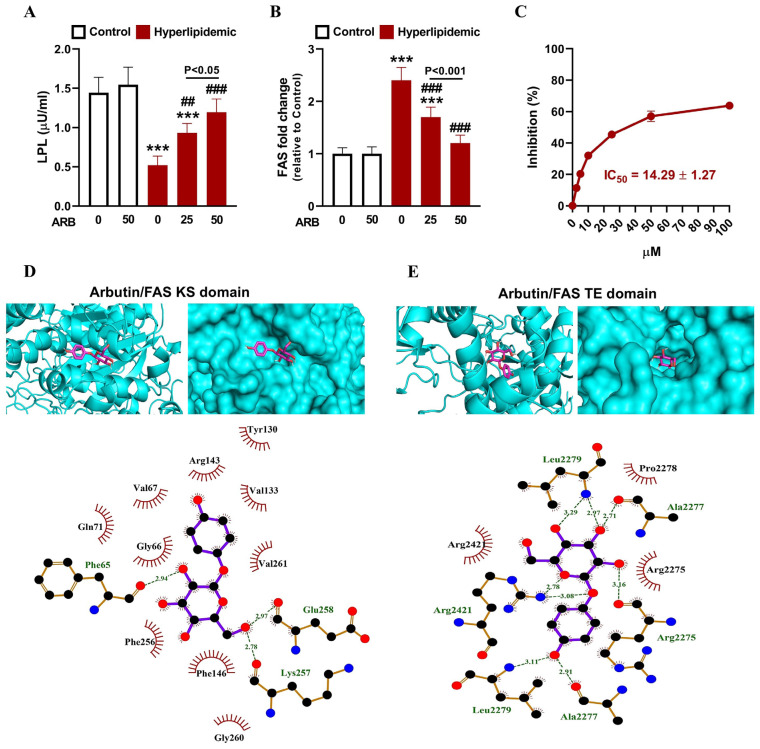
ARB ameliorated plasma LPL activity (**A**) and liver FAS mRNA (**B**) in hyperlipidemic rats. Data are mean ± SEM (*n* = 6). *** *p* < 0.001 vs. Control, and ^##^ *p* < 0.01 and ^###^ *p* < 0.001 vs. Hyperlipidemic. (**C**) In vitro FAS inhibition activity of ARB. Data are mean ± SEM (*N* = 3). (**D**,**E**) Molecular docking of ARB with FAS KS (**D**) and TE (**E**) domains showing the crystal structure and amino acid residues involved in polar bonding and hydrophobic interactions.

Plasma LPL activity was reduced in rats with dyslipidemia (*p* < 0.001), and that effect was reversed dose-dependently by ARB (Figure 5A). In contrast, FAS was upregulated in the liver of rats with dyslipidemia (*p* < 0.001). ARB effectively and dose-dependently ameliorated hepatic FAS in dyslipidemic rats (Figure 5B). The in vitro assay revealed the concentration-dependent FAS inhibitory activity of ARB with an IC_50_ value of 14.29 ± 1.27 µM (Figure 5C). ARB showed the ability to bind the beta-ketoacyl synthase (KS) domain of FAS via polar bonding and hydrophobic interactions with three and ten amino acid residues, respectively (Figure 5D, Table 1), and to form polar bonds and interact hydrophobically with five and three residues of the thioesterase (TE) domain, respectively (Figure 5E, Table 1).

### 2.5. ARB Attenuates Oxidative Stress in Liver of Rats with Dyslipidemia

**Figure 6:**
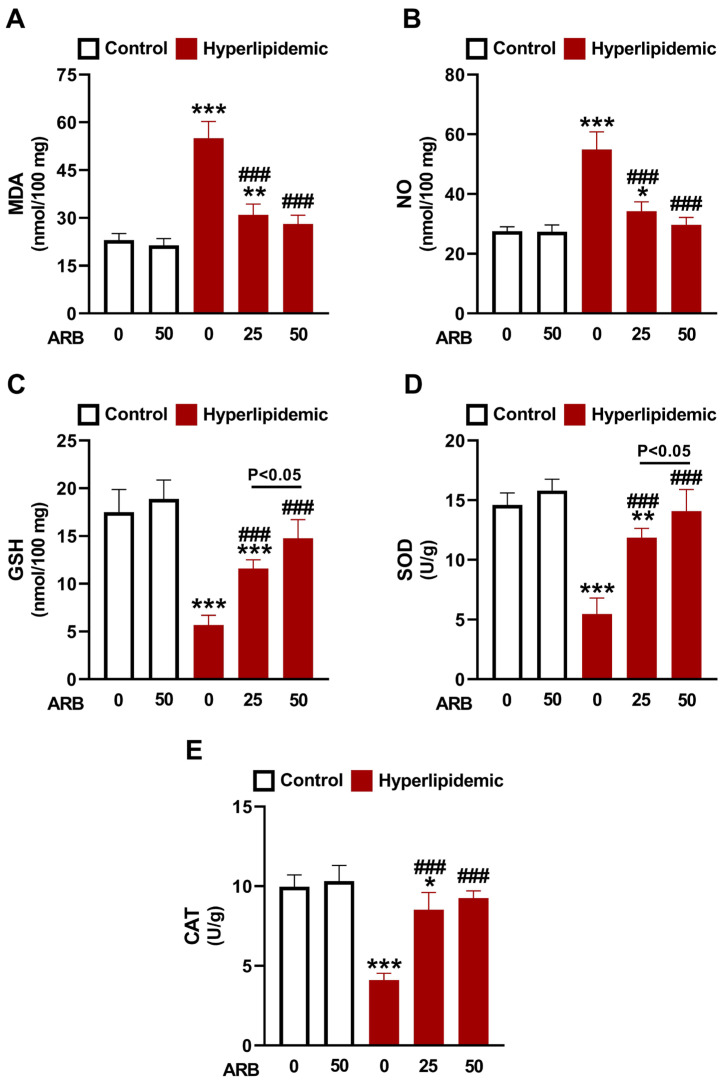
ARB mitigated oxidative stress in hyperlipidemic rats. ARB decreased liver MDA (**A**) and NO (**B**) levels, and enhanced GSH (**C**), SOD (**D**), and CAT (**E**) in hyperlipidemic rats. Data are mean ± SEM (*n* = 6). * *p* < 0.51, ** *p* < 0.01, and *** *p* < 0.001 vs. Control, and ^###^ *p* < 0.001 vs. Hyperlipidemic.

P-407-induced dyslipidemia was associated with elevated liver malondialdehyde (MDA) (Figure 6A) and nitric oxide (NO) (Figure 6B), and a decrease in reduced glutathione (GSH) (Figure 6C), superoxide dismutase (SOD) (Figure 6D) and catalase (CAT) (Figure 6E) (*p* < 0.001). Treatment with either dose of ARB decreased MDA and NO and boosted antioxidants in rats with dyslipidemia. The effect of ARB on GSH and SOD was dose-dependent. Normal rats that received ARB showed no changes in the assayed parameters.

### 2.6. ARB Suppresses Inflammation and Lymphocyte Ecto-Nucleoside Triphosphate Diphosphohydrolase (E-NTPDase) and Ecto-Adenosine Deaminase (E-ADA) in Rats with Dyslipidemia

**Figure 7:**
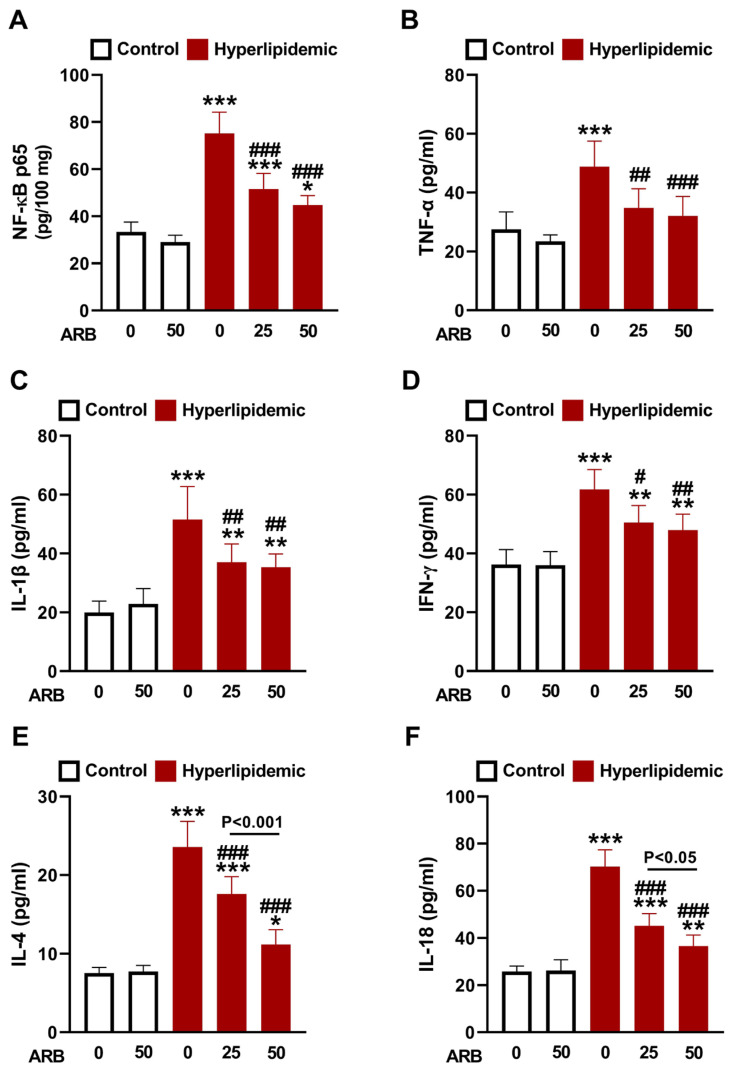
ARB attenuated inflammation in hyperlipidemic rats. ARB suppressed liver NF-κB p65 (**A**) and plasma TNF-α (**B**), IL-1β (**C**), IFN-γ (**D**), IL-4 (**E**), and IL-18 (**F**). Data are mean ± SEM (*n* = 6). * *p* < 0.51, ** *p* < 0.01, and *** *p* < 0.001 vs. Control. ^#^ *p* < 0.05, ^##^ *p* < 0.01, and ^###^ *p* < 0.001 vs. Hyperlipidemic.

**Figure 8:**
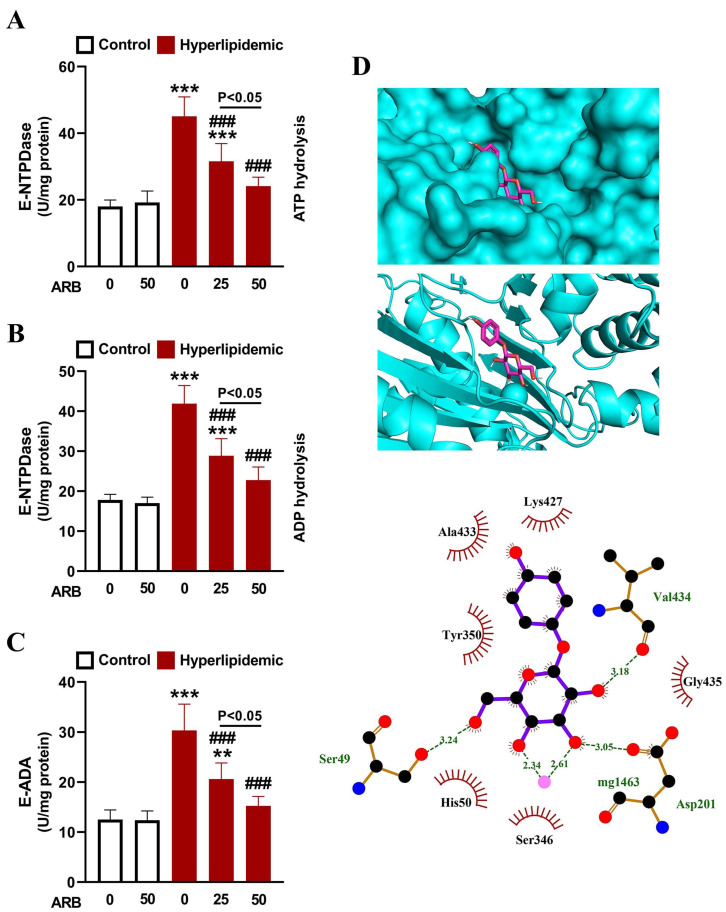
ARB suppressed E-NTPDase (**A**,**B**) and E-ADA (**C**) activities in lymphocytes of hyperlipidemic rats. Data are mean ± SEM (*n* = 6). ** *p* < 0.01 and *** *p* < 0.001 vs. Control, and ^###^ *p* < 0.001 vs. Hyperlipidemic. (**D**) Molecular docking of ARB with E-NTPDase showing the crystal structure and amino acid residues involved in polar bonding and hydrophobic interactions.

To evaluate the effect of ARB on inflammation in rats with dyslipidemia, we measured liver NF-κB p65 that exhibited significant increase in hyperlipidemic rats (*p* < 0.001; Figure 7A). Likewise, circulating TNF-α, IL-1β, IFN-γ, IL-4, and IL-18 were elevated significantly in rats with dyslipidemia as depicted in Figure 7B–F. The activities of lymphocyte E-NTPDase (Figure 8A,B) and E-ADA (Figure 8C) were significantly enhanced in rats with dyslipidemia (*p* < 0.001) as compared with the normal group. ARB showed suppressive effect on lymphocyte both E-NTPDase and E-ADA and its effect was dose-dependent. In addition, ARB exhibited in silico binding affinity with E-NTPDase mediated via three polar bonds and six hydrophobic interactions (Figure 8D, Table 1).

## 3. Discussion

The hydroquinone glucoside ARB exhibited promising health benefits in disorders associated with OS such as diabetes [22,33]. This study showed its beneficial effect against dyslipidemia and its associated OS and inflammatory response using an in vivo animal model, and in vitro and in silico approaches. ARB effectively prevented acute dyslipidemia and its associated OS and inflammation in rats by modulating cholesterologenesis, antioxidants, inflammatory mediators, and lymphocytes E-NTPDase and E-ADA.

The effect of ARB on dyslipidemia was investigated using a P-407-induced rat model. Rats that received P-407 exhibited hypercholesterolemia and hypertriglyceridemia at 12, 24, and 48 h, as reported in previous studies [25,34,35]. In addition to elevated CHOL and TG, LDL and vLDL were elevated, whereas HDL was decreased in plasma of P-407-treated animals. In previous studies, LDL and vLDL were increased, and HDL showed a trend decrease following P-407 injection [25,34,35]. P-407 is a non-toxic and non-ionic copolymer surfactant that is employed to induce dyslipidemia characterized by elevated CHOL and TG [34,35]. P-407 induces dyslipidemia by promoting cholesterologenesis and suppressing TG hydrolysis via LPL inhibition [34,35,36]. Here, plasma LPL was remarkably decreased in rats that received P-407, similarly to previous reports [25,34,35]. The decrease in plasma LPL was conjugated with downregulated liver LDL-R and upregulated HMGCR, demonstrating reduced lipid uptake and increased de novo cholesterologenesis. Through the action of lipase maturation factor 1, LPL is escorted to the endothelial surface where it functions to clear circulating chylomicrons via hydrolysis, and the liberated FAs are taken up by peripheral tissues [37]. LPL hydrolyzes TG within circulating vLDL, which is the main lipoprotein synthesized by the liver and contains TG and CE/CHOL [38]. This hydrolysis releases atherogenic remnant particles with equal content of TG and CE/CHOL [38]. LDL is cleared by liver LDL-R which is continuously recycled [39], and the suppression of LDL-R results in LDL-C elevation, which contributes to atherosclerosis and IHD [1,2]. Hepatocytes have LDL-R on their surface and are central in LDL-C uptake and degradation [40]. LDL-R·LDL complexes are endocytosed into endosomes where the bound lipoproteins are released [41]. With the lipoproteins detached, the LDL-R is recycled back to the plasma membrane [41]. Therefore, the binding of LDL to LDL-R is crucial for reducing circulating LDL; however, this binding could be interrupted by PCSK9, which binds to LDL-R and prevents the binding of LDL [42]. The secreted form of PCSK9 binds to cell surface LDL-R and inhibits its endocytic recycling [43], and the nascent form binds LDL-R and provokes its lysosomal degradation [44]. Hence, the inhibition of PCSK9/LDL-R binding could be valuable for decreasing circulating LDL-C, and this is supported by the LDL-C-lowering efficacy of anti-PCSK9 monoclonal antibodies [45]. The reported decrease in HDL-C demonstrated an atherogenic lipid profile since it is important for decreasing blood CHOL and early atherosclerosis in P-407-administered animals was associated with its low levels [46]. Our findings also showed the involvement of HMGCR upregulation in P-407 dyslipidemia. Rats that received P-407 exhibited an increase in liver HMGCR activity, an effect that could be connected to elevated LDL-C. LDL-R mediated LDL endocytosis downregulates HMGCR expression and upregulate ACAT, leading to the suppression of CHOL biosynthesis [47]. HMGCR is the rate-limiting enzyme in the biosynthesis of CHOL, and its activation is associated with elevated plasma CHOL [6]. The upregulation of HMGCR coincided with previous data showing the association between HMGCR activation and hypercholesterolemia in P-407-treated rats [25]. The elevated levels of CHOL, TG, LDL, and vLDL in P-407-treated rats are directly attributed to the suppression of LPL and LDL-R and upregulation of HMGCR.

ARB effectively prevented dyslipidemia in rats, as shown by the ameliorated TG and CHOL at all checked time points. This amelioration was accompanied by decreased LDL-C and vLDL-C and increased HDL-C, demonstrating the potent anti-dyslipidemia efficacy of ARB. Consistently with this efficacy, we have demonstrated the ability of ARB to ameliorate hyperlipidemia and suppress the atherogenic lipid profile in diabetic rats [22]. The anti-hyperlipidemic effect of ARB in diabetes was attributed to the improved release and sensitivity of insulin, which promotes the uptake of lipids and enhances lipogenesis [48]. The study of Ma et al. attributed the improvement of plasma lipids in mice to the positive effect of ARB on gut development and microbiota [49]. In addition, ARB ameliorated blood CHOL and TG in a rat model of MI, as reported by Sivasangari et al. [50]. Our findings added support to the anti-dyslipidemia efficacy of ARB and introduced novel information on the possible underlying mechanism. ARB increased plasma LPL activity and upregulated liver LDL-R resulting in TG hydrolysis and LDL-C uptake, respectively. The positive effect of ARB on LDL-R and consequently LDL-C uptake was further supported by in silico findings showing the affinity of ARB to bind the binding domain of PCSK9 on LDL-R through polar and hydrophobic interactions. This binding affinity might have a role in preventing the binding of PCSK9 to LDL-R and degradation of the latter and allowing its recycling and uptake of LDL-C. Moreover, the decrease in CHOL could be directly attributed to the inhibition of HMGCR and suppression of de novo cholesterologenesis. HMGCR inhibitory activity of ARB was further confirmed using in vitro and in silico data. In vitro, ARB exhibited concentration-dependent inhibition of HMGCR, findings that were supported by the binding affinity of ARB towards many amino acid residues in the enzyme binding site. Besides HMGCR suppression, ARB-mediated modulation of ABCA1 expression might be involved in CHOL homeostasis and elevated HDL-C levels. ABCA1 plays a key role in RCT and maintenance of CHOL homeostasis [12]. ABCA1 contributes to the synthesis of HDL-C by exporting phosphatidylcholine and CHOL to the circulating lipid-free ApoA-I [12]. Pre-clinical studies on specific knockout murine models revealed that ABCA1-mediated biogenesis of HDL within the liver constitutes 70% of the total generated HDL [51]. Interestingly, ARB upregulated ABCA1 expression, explaining at least in part its beneficial role on CHOL homeostasis and the improved HDL-C levels. Of note, neither ABCG5 nor ABCG8 were affected by P-407 with and without ARB. These findings were consistent with our and others previous data [16,25,36]. ABCG5 and ABCG8 are involved in the secretion of CHOL in the liver and intestine [11]. Accordingly, the amelioration of cholesterologenesis by ARB involved the suppression of CHOL biogenesis and enhancement of HDL production, with no effect on hepatobiliary and trans-intestinal CHOL secretion. ARB-mediated the suppression of de novo cholesterologenesis, and dyslipidemia was further confirmed by its ability to inhibit FAS. FAS is a homodimeric multidomain enzyme that produces palmitate using acetyl-CoA and malonyl-CoA [52]. The synthesis of palmitate and subsequent TG represent the process of lipogenesis. Palmitate is incorporated into TG and acts as a precursor in the synthesis CHOL and other complex lipids [53]. Therefore, the inhibition of FAS activity can contribute to the suppression of TG and CHOL synthesis. Here, ARB dose-dependently suppressed FAS activity both in dyslipidemic rats in vivo and in vitro. This experimentally validated inhibitory effect was supported by in silico findings showing the strong binding affinity of ARB towards KS and TE domains of FAS.

Owing to the growing evidence showing the relationship between dyslipidemia and OS and inflammation [13,18], we assumed that the antioxidant and anti-inflammatory activities of ARB contribute to its beneficial role against dyslipidemia. In this study, MDA and NO were elevated in rats with dyslipidemia, whereas GSH and antioxidant enzymes were decreased. These findings demonstrated OS as we previously reported in the same model [16,25]. Redox imbalance and dyslipidemia are implicated in CVDs such as atherosclerosis [13,16]. Besides damage to cellular macromolecules and cell injury, elevated ROS in dyslipidemia oxidize LDL resulting in endothelial cell activation and recruitment of monocytes and T lymphocytes [54]. ROS also activate NF-κB and cytokine release, effects reported in this study where P-407-treated rats exhibited upregulation of liver NF-κBp65 and circulating TNF-α, IL-1β, IFN-γ, IL-4, and IL-18. In addition, dysregulated lymphocyte E-NTPDase and E-ADA activities under dyslipidemia contribute to the pro-inflammatory response [25]. High extracellular ATP levels promote pro-inflammatory cytokine release from lymphocytes [55]. ATP is hydrolyzed by E-NTPDase on the surface of immune cells, and the generated AMP is further hydrolyzed by E-5′-nucleotidase. E-ADA deaminates the generated adenosine resulting in proliferation and differentiation of monocytes and T lymphocytes and the development of an inflammatory response [55]. Therefore, dyslipidemia is a key factor in chronic inflammation promoted by disrupted leukocyte activity and cytokine regulation [56]. Elevated CHOL, LDL-C, and TG have been reported to activate E-NTPDase and E-ADA and promote cytokine release in rodents [57,58,59]. The released cytokines are implicated in the development of CVD, including atherosclerosis. IL-4 and IFN-γ are pro-atherogenic cytokines with a significant role in atherosclerosis [60,61]. For instance, IFN-γ provokes the adhesion of leukocytes to the endothelial lining of blood vessels [60,61]. IL-18 is a pro-inflammatory mediator implicated in the development and progression of atherosclerotic plaque [62], and TNF-α, IL-6, and IL-1β are pro-inflammatory cytokines with a role in disrupting insulin action and lipid storage and their levels were reported to elevate in different tissues of P-407-induced dyslipidemic rats [63,64].

ARB attenuated OS, enhanced antioxidant defenses and mitigated the inflammatory response associated with dyslipidemia. The effects of ARB included the suppression of lymphocyte E-NTPDase and E-ADA activities. The reported dual antioxidant and anti-inflammatory efficacy of ARB was supported by previous studies showing its ability to attenuate OS and inflammation in diabetes and other disorders [22,31,33,65,66]. In animal models of diabetes, lung injury, cardiac hypertrophy, and acute kidney damage, ARB boosted antioxidants and suppressed inflammation [31,33,65,66]. In addition to these studies, our investigation added new information on the anti-inflammatory mechanism of ARB. In view of the significant role of E-NTPDase and E-ADA in inflammatory response and cytokine release, the suppression of the activities of these enzymes might have contributed to the anti-inflammatory efficacy of ARB in rats with dyslipidemia. The findings of molecular docking revealed the affinity of RB to bind several E-NTPDase amino acid residues, adding support to its anti-inflammatory role.

## 4. Materials and Methods

### 4.1. Experimental Design

**Figure 9:**
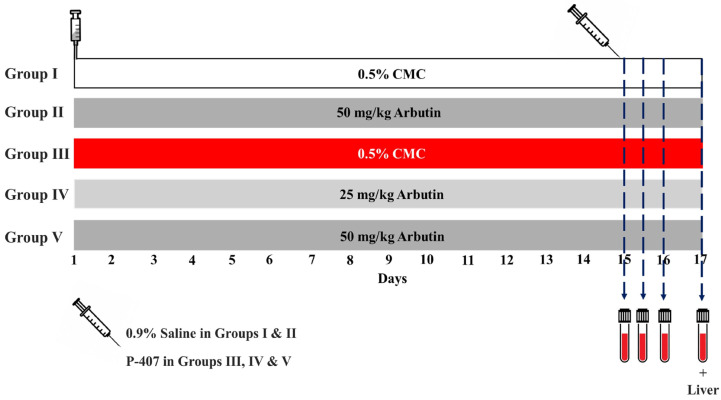
A schematic diagram of the experimental groups and treatments.

Thirty adult male Wistar rats (160–180 g) were kept under standard conditions (temperature 23 ± 1 °C and humidity 50–60%) and provided standard pellet food and water *ad libitum*. To investigate the anti-hyperlipidemic effect of ARB, 500 mg/kg P-407 (Sigma, St. Louis, MO, USA) was injected intraperitoneally (i.p.) to induce dyslipidemia [16,67] and control rats received physiological saline. The rats were divided into five groups, two normal groups (I and II) and three dyslipidemic groups (III, IV and V), each with six rats (*n* = 6), as outlined below (Figure 9):

Group I: received 0.5% carboxymethyl cellulose (CMC) via oral gavage for 14 days.
Group II: received 50 mg/kg ARB (Sigma, St. Louis, MO, USA) [22] suspended in 0.5% CMC via oral gavage for 14 days.
Group III: received 0.5% CMC via oral gavage for 14 days.
Group IV: received 25 mg/kg ARB [22] in 0.5% CMC via oral gavage for 14 days.
Group V: received 50 mg/kg ARB [22] in 0.5% CMC via oral gavage for 14 days.

P-407 was administered on day 15, and blood samples were collected from tail vein before P-407 and at 12, 24, and 48 h for the assay of TG and CHOL. After 48 h, the rats were anesthetized with ketamine and xylazine administered i.p. at doses of 100 mg/kg and 10 mg/kg, respectively, sacrificed and liver samples were collected. Liver samples were homogenized (10% *w*/*v*) in Tris-HCl buffer (pH 7.4) and the homogenate was centrifuged, and the clear supernatant was collected.

### 4.2. Biochemical Assays

Plasma CHOL, HDL-C and TG were assayed using Biosystems kits (Barcelona, Spain; Cat. no.: 21505, 11523, and 11528, respectively), and vLDL and LDL were calculated as following:


*LDL = Total Cholesterol − (HDL + vLDL)*

LPL, NF-κB p65, and cytokines (IFN-γ, IL-4, IL-1β, TNF-α, and IL-18) were measured using Solarbio (Beijing, China; Cat. no. BC2440) and ELabscience (Wuhan, China; Cat. no.: E-EL-R0009, E-EL-R0014, E-EL-R0012, E-EL-R2856, and E-EL-R0567, respectively) kits, respectively. MDA, NO, GSH, SOD, and catalase were determined using Bio-Diagnostic kits (Giza, Egypt; Cat. no.: MD2528, NO2533, TA2511, SD2521, and CA2517, respectively) kits. HMGCR activity was determined in the liver of rats by measuring the ratio of HMG-CoA to mevalonate as previously described [68]. In vitro HMGCR inhibition assay was performed as previously described by Wang et al. [69] using different concentrations of ARB and ATOR and monitoring the consumption of NADPH. The inhibitory activity of ARB against FAS was determined following the method of Jiang et al. [70] by monitoring NADPH consumption at 340 nm. To determine the activities of E-NTPDase and E-ADA, lymphocyte-rich mononuclear cells were isolated from blood on EDTA using Ficoll-Histopaque (Sigma, St. Louis, MO, USA) density-gradient centrifugation [71]. Trypan blue exclusion was employed to check the integrity of lymphocytes [72]. Following treatment with 1% Triton X-100, Bradford reagent [73] was used to determine protein content. The activities of E-NTPDase and E-ADA were assayed according to Leal et al. [74] and Giusti and Galanti [75], respectively. To determine E-NTPDase activity, 200 μL of a reaction mixture containing 0.5 mM CaCl_2_, 120 mM NaCl, 60 mM glucose, 5 mM KCl, and 50 mM Tris–HCl buffer (pH 8.0) was added to 20 μL of the cell suspension preincubated for 10 min at 37 °C. The reaction was initiated by the addition of ATP or ADP (2 mM final concentration), and 200 μL of 10% trichloroacetic acid was added to stop the reaction. The concentration of inorganic phosphate released during the reaction was measured using malachite green as a colorimetric reagent and potassium dihydrogen phosphate as a standard. The absorbance of the reaction was determined at 630 nm [76]. To assess E-ADA activity, 21 mM adenosine was added to 25 μL of the cell preparation. Following incubation at 37 °C for 1 h, sodium nitroprusside and hypochlorite solution was added to stop the reaction. Ammonium sulfate (75 μM) was used as a standard.

### 4.3. qRT-PCR

Changes in liver LDL-R, ABCA1, ABCG5, ABCG8, and FAS mRNA were assessed using qRT-PCR as previously described [25]. Briefly, total RNA was isolated using Trizol (Invitrogen, ThermoFisher Scientific, Waltham, MA, USA; Cat. no.: 15596026) and RNA samples showed OD260/OD280 value ≥ 1.8 after purification were reverse transcribed into cDNA. Amplification of DNA was performed using SYBR Green Master Mix (ThermoFisher Scientific, Waltham, MA, USA; Cat. no.: 4309155) and the primer pairs in Supplementary Table S1. The 2^−ΔΔCt^ method [77] was employed for analysis using β-actin as a control.

### 4.4. In Silico Molecular Docking

The affinity of ARB towards HMGCR (PDB: 1DQA), LDL-R PCSK9 binding domain (PDB: 3GCX), FAS KS domain (PDB: 3HHD), FAS TE domain (PDB: 1XKT), and E-NTPDase (PDB: 4BQZ) was investigated using PyRx virtual screening software (version 0.8) [78], which uses AutoDock Vina as a docking software platform. For molecular docking, Autodock Vina was employed with a grid box centered on the active sites of the target proteins. The grid box dimensions were set to encompass the binding site adequately to allow for flexible docking. The exhaustiveness parameter, which controls the thoroughness of the search, was set to 8, and a total of 10 conformations were generated for each ligand–protein complex. Autodock Tools (ADT; v1.5.6) was used for the preparation of the target proteins which included the removal of water molecules, addition of polar hydrogens, and assignment of Gasteiger charges. Ligands were prepared by optimizing their geometry and assigning appropriate torsional degrees of freedom. The selection of the optimal docked conformation was based on the lowest binding energy, as well as visual inspection of interactions within the binding site. The PyMOL software (v2.3.2) was used to visualize the binding modes, while LigPlot (v2.2.8) [79] was employed to analyze the protein-ligand interactions, highlighting key hydrogen bonds and hydrophobic contacts between the ligand and target proteins.

### 4.5. Statistical Analysis

The data are represented as mean ± SEM. Analysis of the statistical differences between groups was carried out using one-way ANOVA followed by Tukey’s test on GraphPad 8. A *p* value < 0.05 was considered significant.

## 5. Conclusions

These findings demonstrated the efficacy of ARB to prevent acute dyslipidemia and its associated OS and inflammation in rats. ARB effectively suppressed HMGCR and FAS, and upregulated LPL, LDL-R, and ABCA1, resulting in suppressed cholesterologenesis and enhanced TG hydrolysis. In addition, ARB attenuated OS, lymphocyte E-NTPDase and E-ADA and inflammatory response, and boosted antioxidants in dyslipidemic rats. ARB showed in silico affinity to bind LDL-R PSK9 binding domain, HMGCR, FAS, and E-NTPDase. Thus, ARB effectively ameliorated acute dyslipidemia, OS, and inflammation by modulating cholesterologenesis and TG hydrolysis.

## Figures and Tables

**Table 1:** Binding affinities of ARB towards LDL-R PCSK9 binding domain, HMGCR, FAS, and E-NTPDase.

	Binding Energy (kcal/mol)	Polar Interacting Residues	Hydrophobic Interacting Residues
LDL-R PCSK9 binding domain	−5.8	Tyr166, Ala168, Tyr171, Val241	Ala242, Lys243, Ala397, Ser401, Val180, Met398, Tyr166, Ala245
HMGCR	−7.7	Gln766, Val805, Gly765, Met655, Asn658	Thr809, Gly807, Asp767, Glu559, Ala654, Gly806, Gly803, His866
FAS KS	−6.5	Glu258, Lys257, Phe256	Gln71, Phe65, Val67, Arg143, Tyr130, Val133, Gly66, Val261, Phe146, Gly260
FAS TE	−8.0	Leu2279, Arg2421, Arg2275, Ala2277, Leu2279, Ala2277	Pro2278, Arg2275, Arg242
E-NTPDase	−6.5	Val434, Asp201, Ser49	Ala433, Lys427, Tyr350, Gly435, His50, Ser346

LDL-R PCSK9: low-density lipoprotein receptor proprotein convertase subtilisin/kexin type 9; HMGCR: 3-hydroxy-3-methylglutaryl CoA reductase; FAS: fatty acid synthase; KS; beta-ketoacyl synthase; TE: thioesterase; E-NTPDase: ecto-nucleoside triphosphate diphosphohydrolase.

## Data Availability

The manuscript and Supplementary Materials contain all data supporting the reported results.

## Supplementary Materials

The following supporting information can be downloaded at: https://www.mdpi.com/article/10.3390/ph17101343/s1, Table S1: Primers used for qRT-PCR.
