# Supplementary material for: Oxidative Stress, Inflammation, and Altered Lymphocyte E-NTPDase Are Implicated in Acute Dyslipidemia in Rats: Protective Role of Arbutin
Source: Pharmaceuticals (Basel). 2024 Oct 8;17(10):1343. doi: 10.3390/ph17101343 (PMC11509952; doi:10.3390/ph17101343)
Supplement: Supplementary file 1 [file pharmaceuticals-17-01343-s001.zip › pharmaceuticals-3223145-supplementary.pdf]

Supplementary Table S1. Primers used for qRT-PCR.

Gene	Genbank accession number	Sequence (5'-3')	Amplicon size (bp)
Abca1	NM_178095.3	F: GCAGCGACCATGAAAGTGAC<br>R: GAGGCGGTCATCAATCTCGT	185
Abcg5	NM_053754.2	F: GGGAAGTGTTTGTGAACGGC<br>R: GTGTATCTCAGCGTCTCCCG	121
Abcg8	NM_130414.2	F: TTCTGATGACGTCTGGCACC<br>R: TTGCTGTAGCGAGACAAGG	97
Ldlr	NM_175762.3	F: CATTTTCAGTGCCAACCGCC<br>R: TGCCTCACACCAGTTTACCC	127
FASN	NM_017332.2	F: CTGGACTCGCTCATGGGTG<br>R: CATTTCCTGAAGCTTCCGCAG	111
Actb	NM_031144.3	F: AGGAGTACGATGAGTCCGGC<br>R: CGCAGCTCAGTAACAGTCCG	71
